# Structural insights into histone chaperone Chz1-mediated H2A.Z recognition and histone replacement

**DOI:** 10.1371/journal.pbio.3000277

**Published:** 2019-05-20

**Authors:** Yunyun Wang, Sheng Liu, Lu Sun, Ning Xu, Shan Shan, Fei Wu, Xiaoping Liang, Yingzi Huang, Ed Luk, Carl Wu, Zheng Zhou

**Affiliations:** 1 National Laboratory of Biomacromolecules, CAS Center for Excellence in Biomacromolecules, Institute of Biophysics, Chinese Academy of Sciences, Beijing, China; 2 University of Chinese Academy of Sciences, Beijing, China; 3 Department of Biology, Johns Hopkins University, Baltimore, Maryland, United States of America; 4 Department of Biochemistry and Cell Biology, Stony Brook University, Stony Brook, New York, United States of America; National Cancer Institute, UNITED STATES

## Abstract

Chz1 is a specific chaperone for the histone variant H2A.Z in budding yeast. The ternary complex formed by Chz1 and H2A.Z-H2B dimer is the major in vivo substrate of Swi2/snif2-related 1 (SWR1), the ATP-dependent chromatin remodeling enzyme that deposits H2A.Z into chromatin. However, the structural basis for the binding preference of Chz1 for H2A.Z over H2A and the mechanism by which Chz1 modulates the histone replacement remain elusive. Here, we show that Chz1 utilizes 2 distinct structural domains to engage the H2A.Z-H2B dimer for optimal and specific recognition of H2A.Z. The middle region of Chz1 (Chz1-M) directly interacts with 2 highly conserved H2A.Z-specific residues (Gly98 and Ala57) and dictates a modest preference for H2A.Z-H2B. In addition, structural and biochemical analysis show that the C-terminal region of Chz1 (Chz1-C) harbors a conserved DEF/Y motif, which reflects the consecutive D/E residues followed by a single aromatic residue, to engage an arginine finger and a hydrophobic pocket in H2A.Z-H2B, enhancing the binding preference for H2A.Z-H2B. Furthermore, Chz1 facilitates SWR1-mediated H2A.Z deposition by alleviating inhibition caused by aggregation of excess free histones, providing insights into how Chz1 controls the bioavailability of H2A.Z to assist SWR1 in promoter-specific installation of a histone mark. Our study elucidates a novel H2A.Z-recognition mechanism and uncovers a molecular rationale for binding of free histone by specialized histone chaperones in vivo.

## Abstract 2

The variant histone H2A.Z is essential for the viability of metazoans. The structure of H2A.Z-H2B histone dimer in complex with the chaperone Chz1 reveals the basis of Chz1’s specificity for recognising H2A.Z. In vitro studies show that Chz1 facilitates H2A.Z deposition by relieving the inhibitory effects of excess free histone dimers.

## Introduction

In eukaryotes, genetic materials are packaged into nucleosome composed of histones and DNA. The canonical nucleosome is formed by 147 base pair of DNA wrapping around a histone octamer, which contains 2 copies of histone H2A, H2B, H3, and H4 [1]. Incorporation of histone variants results in noncanonical nucleosomes with altered structure and function [2]. H2A.Z is an evolutionarily conserved histone variant of H2A and is essential for the viability of metazoans [3]. H2A.Z plays important roles in a number of biological activities, including accurate transcriptional response, maintenance of genome integrity, and chromosome transmission fidelity [4].

Chromatin incorporation of H2A.Z is catalyzed by the Swi2/snif2-related 1 (SWR1) complex in yeast and the Snf2-related CBP activator protein (SRCAP) and the Protein 400 (p400)/ 60kDa Tat-interactive protein (Tip60) complexes in mammals, which are ATP-dependent chromatin remodelers [5–8]. H2A.Z incorporation occurs via an ATP-driven histone exchange reaction by which each of the 2 H2A-H2B (A-B) dimers of a canonical nucleosome is replaced with 2 H2A.Z-H2B (Z-B) dimers in a stepwise manner[6,9].

The SWR complex protein 2 (Swc2; Swc2 homolog in higher eukaryotes [YL1] in higher eukaryotes) and Swr1 subunits of the SWR1 remodeler contain specific Z-B binding motifs that are required for efficient substrate binding and optimal H2A.Z deposition activity [10–12]. These Z-B binding motifs are highly enriched for acidic residues and are predicted to be unstructured in the unbound state [10,12]. Upon binding, induced folding occurs such that the acidic motifs wrap around basic regions on the Z-B dimer used to interact with DNA in the context of nucleosome [10,12]. This appears to be a common mechanism for other interacting proteins of unincorporated Z-B dimers.

How histone chaperones preferentially recognize Z-B dimers over A-B dimers is a central theme of this study. Previously, we and others found that acidic leucine-rich nuclear phosphoprotein 32 family member e (Anp32e), a mammal-exclusive H2A.Z chaperone, is involved in the disassembly of H2A.Z-containing nucleosomes [13,14]. Structural studies showed that Anp32e recognizes Z-B dimer via interaction with the alpha helix C terminal (αC helix) of H2A.Z, which is 1 amino acid shorter than the corresponding region of H2A [13,14]. The missing residue is a glycine, an alpha helix-destabilizing amino acid. As such, H2A.Z in its Z-B dimeric form has an extended αC helix, thereby providing additional contacts with Anp32e [13]. A similar recognition mechanism has been observed for YL1 and Swr1 in their preferential binding of Z-B [10,12].

Chz1 is a major chaperone for H2A.Z in yeast and has several reported nuclear functions. For example, it is involved in nuclear import of H2A.Z [15] and transcription of subtelomeric genes [16]. But the best characterized function of Chz1 is its role in H2A.Z deposition. Chz1 forms a trimeric complex with Z-B dimer and cooperates with SWR1 in histone exchange [17,18]. Chz1 preferentially binds to the Z-B dimer over the A-B dimer [17]. One hypothesis is that Chz1 is the first leg of a Z-B dimer relay that delivers the histone substrate to Swc2 and Swr1, which in turn insert the dimer into nucleosome. Chz1 binds to the bulk of Z-B dimers in vivo. But the histone chaperones Nucleosome assembly protein 1 (Nap1) and Facilitates chromatin transcription (FACT) can also bind to Z-B dimers [17]. Moreover, multiple histone chaperones function redundantly to capture most, if not all, free Z-B dimers inside the cell [17]. Why it is necessary to minimize the cellular level of free Z-B dimers is unclear.

Chz1 is unstructured in the unbound state [19]. The NMR structure of the Chz1–Z-B ternary complex showed that the middle region (amino acid 64–124) of Chz1 (termed Chz1-M) interacts with the Z-B dimer via both acidic and basic residues to dock onto complementary sites on the histone substrate [19]. However, in contrast to the aforementioned H2A.Z binding motifs, Chz1-M does not recognize the extended αC helix of H2A.Z. Therefore, what structural determinants Chz1 uses to distinguish H2A.Z from H2A remain elusive.

In this study, we systematically analyzed structural elements of Chz1 and H2A.Z that dictate binding specificity. Analysis of the binding between Chz1-M and H2A.Z-H2B dimer revealed 2 highly conserved H2A.Z-specific residues that confer H2A.Z the preference. Moreover, we showed that a previously uncharacterized C-terminal region of Chz1 (Chz1-C) is required for optimal Z-B recognition in vitro and H2A.Z incorporation in vivo and solved a 1.65-Å crystal structure of Z-B dimer in complex with Chz1-C. Finally, we found that Chz1 facilitates SWR1-catalyzed H2A.Z deposition in vitro by relieving the inhibitory effects of excess free Z-B dimers, which impedes the reaction by forming histone–DNA aggregates. Overall, this study shows how Chz1 utilizes 2 binding sites to cooperatively recognize the Z-B dimer, ensuring the bioavailability of the histone substrate of SWR1.

## Results

### Characterization of full-length Chz1 for H2A.Z-H2B binding

**Fig 1:**
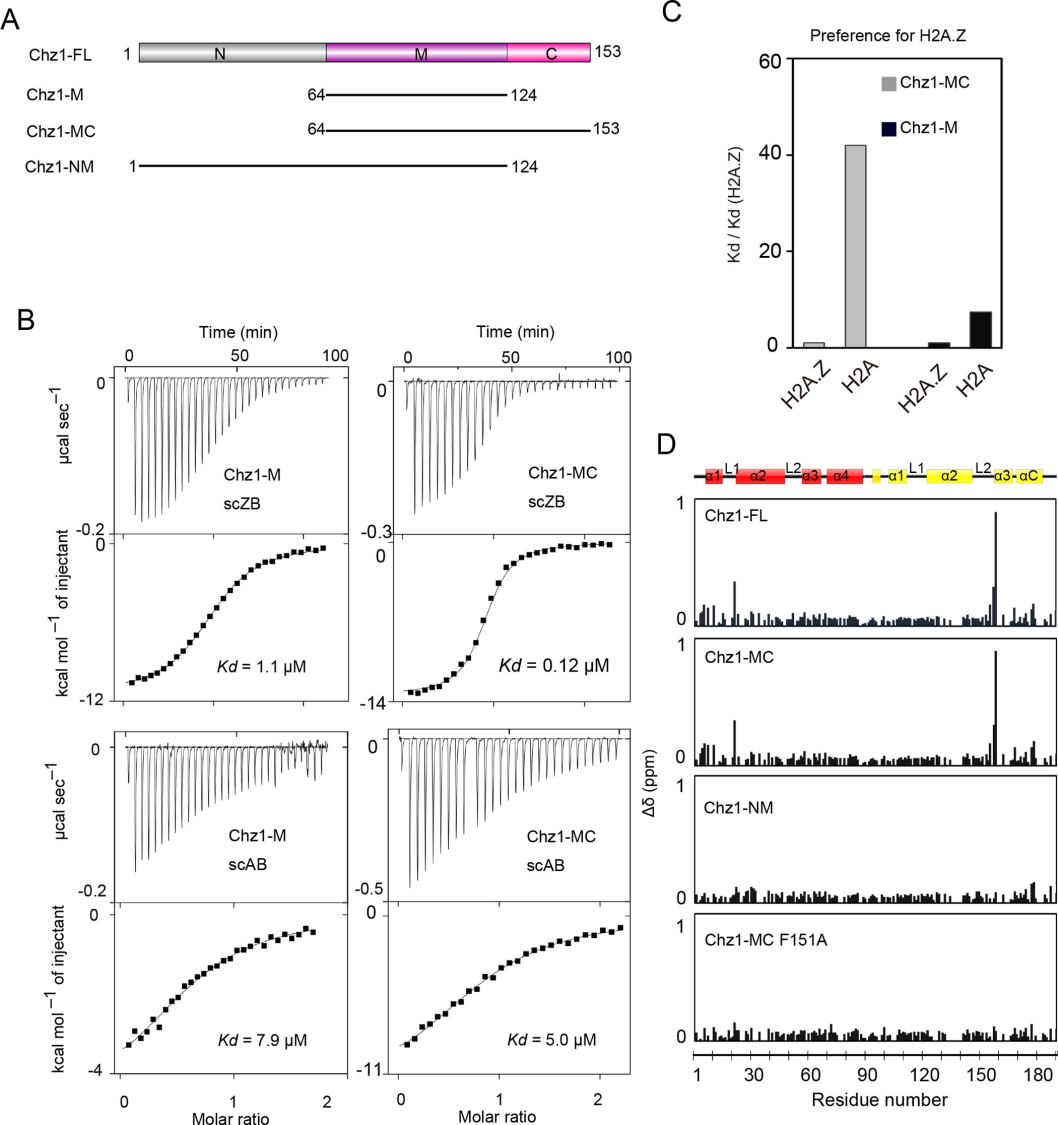
Identification of Chz1 regions dictates the preference for H2A.Z. (A) Schematic view of Chz1-FL according to a previous mapping result. Shown below the scheme include the N-terminal region (residues 1–63), middle region (residues 64–124), and C-terminal region (residues 125–153) of Chz1 termed Chz1-N, Chz1-M, and Chz1-C, respectively. (B) Effect of Chz1-MC and Chz1-M on the binding of H2A.Z-H2B dimer and H2A-H2B dimer by ITC. Histone dimers were titrated by Chz1-M (left) and Chz1-MC (right). The underlying data can be found in S1 Data. (C) Effect of Chz1-MC and Chz1-M on dictating the preference for H2A.Z. The folds of binding affinity decrease between all histone dimers (in single-chain form) and scZB are denoted as Kd/Kd (H2A.Z) and calculated for comparison. The underlying data can be found in S1 Data. (D) CSP mapping analysis of H2A.Z-H2B residues interacting with different Chz1. The ^1^H-^15^N HSQC spectrum of ^15^N labeled scZB in complex with Chz1-M serves as the reference spectra to monitor the CSP changes. The CSP changes are calculated as Δδ = [(Δδ^1^H)^2^ + (Δδ^15^N/5)^2^]^1/2^ and plotted as a function of scZB residues for comparison. From the top to bottom: CSP changes of scZB residues interacting with Chz1-FL, Chz1-MC, Chz1-NM, Chz1 F151A mutant. On the top is displayed the schematic view of scZB and secondary structures of H2A.Z and H2B, which are colored in yellow and red, respectively. The underlying data can be found in S2 Data. Chz1, chaperone for H2A.Z-H2B; Chz1-C, C-terminal region of Chz1; Chz1-FL, full-length Chz1; Chz1-M, middle region of Chz1; Chz1-MC, middle and C-terminal region of Chz1; Chz1-N, N-terminal region of Chz1; Chz1-NM, N-terminal and middle region of Chz1; CSP, chemical shift perturbation; HSQC, heteronuclear single-quantum coherence; ITC, isothermal titration calorimetry; scAB, single-chain H2A-H2B; scZB, single-chain H2A.Z-H2B.

The full-length Chz1 (Chz1-FL) is composed of an N-terminal region (Chz1_1–63_, Chz1-N), a middle region (Chz1_64–124_, termed Chz1-M), and a C-terminal region (Chz1_125–153_, termed Chz1-C; Fig 1A). A previous study has shown that Chz1-M forms a stable complex with yeast single-chain H2A.Z-H2B (scZB) [19]. To investigate whether Chz1 exhibits a binding preference for H2A.Z, we performed isothermal titration calorimetry (ITC) to analyze the binding between different Chz1 proteins and scZB or yeast single chain H2A-H2B (scAB; Fig 1B, Table 1). We find Chz1-M achieves an approximately 7.5-fold higher affinity for scZB (*Kd* of 1.06 μM) than for scAB (*Kd* of 7.9 μM), presenting a modest binding preference for H2A.Z (Fig 1B). The binding between scZB and Chz1-MC (Chz1_64–153_) (*Kd* = 0.12 μM) or Chz1-FL (*Kd* = 0.13 μM) results in remarkably higher affinities than the binding between scZB and Chz1-M (Fig 1B, Table 1, compare rows 1–3). As a consequence, Chz1-MC achieves an approximately 42-fold higher affinity for scZB (*Kd* of 0.12 μM) than for scAB (*Kd* of 5.0 μM), presenting an optimal specificity for H2A.Z (Fig 1C). The increased affinity between H2A.Z and Chz1-MC or Chz1-FL strongly suggests that Chz1-C may contain an uncovered scZB binding region that might enhance the interaction between Chz1-MC and scZB.

**Table 1:** Comparison of the binding affinities measured by ITC.

Sample and buffer condition for ITC analysis				*N* (sites)	*Kd* (μM)	Number
Chz1 (0.5 M NaCl)	Chz1-FL	H2A.Z	H2B	1.0	0.13 ± 0.001	1
	Chz1-M			1.0	1.06 ± 0.04	2
	Chz1-MC			0.9	0.12 ± 0.01	3
Chz1-MC (0.5 M NaCl)	H2A.Z	WT	H2B	0.9	0.12 ± 0.01	4
	H2A.Z	A57P/G98N	H2B	1.1	3.39 ± 0.21	5
	H2A.Z	G98N	H2B	0.9	0.97 ± 0.03	6
	H2A.Z	A57P	H2B	1.0	0.74 ± 0.03	7
	H2A.Z	RA→GNV	H2B	1.0	0.19 ± 0.01	8
	H2A	WT	H2B	0.9	5.02 ± 0.42	9
	H2A	P50A/N91G	H2B	1.1	0.16 ± 0.01	10
	H2A	P50A	H2B	1.0	0.53 ± 0.04	11
	H2A	N91G	H2B	1.0	0.42 ± 0.02	12
	H2A	NKLGNV→DSIRA	H2B	0.9	5.08 ± 0.18	13
	H2A.Z	H2B	Y45A/M62A	0.8	1.12 ± 0.01	14
Chz1-M (0.5 M NaCl)	H2A.Z	WT	H2B	1.0	1.06 ± 0.04	15
	H2A	WT		0.7	7.87 ± 0.35	16
Swc2-Z (0.2 M NaCl)	H2A.Z	WT	H2B	1.1	0.04 ± 0.004	17
		A57P/G98N		0.8	0.04 ± 0.005	18
	H2A	WT	H2B	0.9	0.20 ± 0.07	19
		P50A/N91G		1.0	0.17 ± 0.02	20
Chz1-M (0.2 M NaCl)	H2A.Z	WT	H2B	1.0	0.02 ± 0.002	21
	H2A	WT		1.0	0.30 ± 0.002	22

**Abbreviations:** Chz1, chaperone for H2A.Z-H2B; Chz1-M, middle region of Chz1; Chz1-MC, middle and C-terminal region of Chz1; ITC, isothermal titration calorimetry; Swc2, SWR complex protein 2; Swc2-Z, H2A.Z binding domain of Swc2; WT, wild type

We next performed NMR chemical shift perturbation (CSP) mapping, asking which residues in Z-B dimer interact with Chz1 (Fig 1D). Comparison of the ^1^H–^15^N heteronuclear single-quantum coherence (HSQC) spectra of the ^15^N labeled scZB in complex with nonlabeled Chz1-FL and Chz1-M show large chemical shift changes, indicating that the Chz1 residues outside the Chz1-M region are involved in scZB binding (S1 Fig). We next compared the ^1^H–^15^N HSQC spectra of scZB in complex with Chz1-FL, Chz1-MC, and N-terminal and middle region of Chz1 (Chz1-NM) with the spectra of scZB in complex with Chz1-M (Fig 1D). We calculate the changes of CSP (Δδ) and generate CSP profiles for Chz1-FL, Chz1-MC, and Chz1-NM by plotting the Δδ plotted along the protein sequence of scZB (Fig 1D). The CSP profile of Chz1-NM displays a minimal change of CSP, suggesting that the scZB binding adopted by Chz1-NM and Chz1-M are similar. In contrast, the large CSP changes observed in CSP profiles of Chz1-FL and Chz1-MC suggest that in addition to scZB residues interacting with Chz1-M, other scZB residues are recognized by these 2 Chz1 proteins (Fig 1D). We conclude that residues in H2A.Z L2 loop, H2B L1 loop, and H2B α1 and α2 helices are involved in these additional interactions, given that the large CSP changes are exclusively observed in these regions (Fig 1D). These findings also suggest that the Chz1-C region, rather than the Chz1-N region, mediates the additional interactions with scZB.

### Structural basis for H2A.Z-H2B dimer recognition by Chz1-C

**Fig 2:**
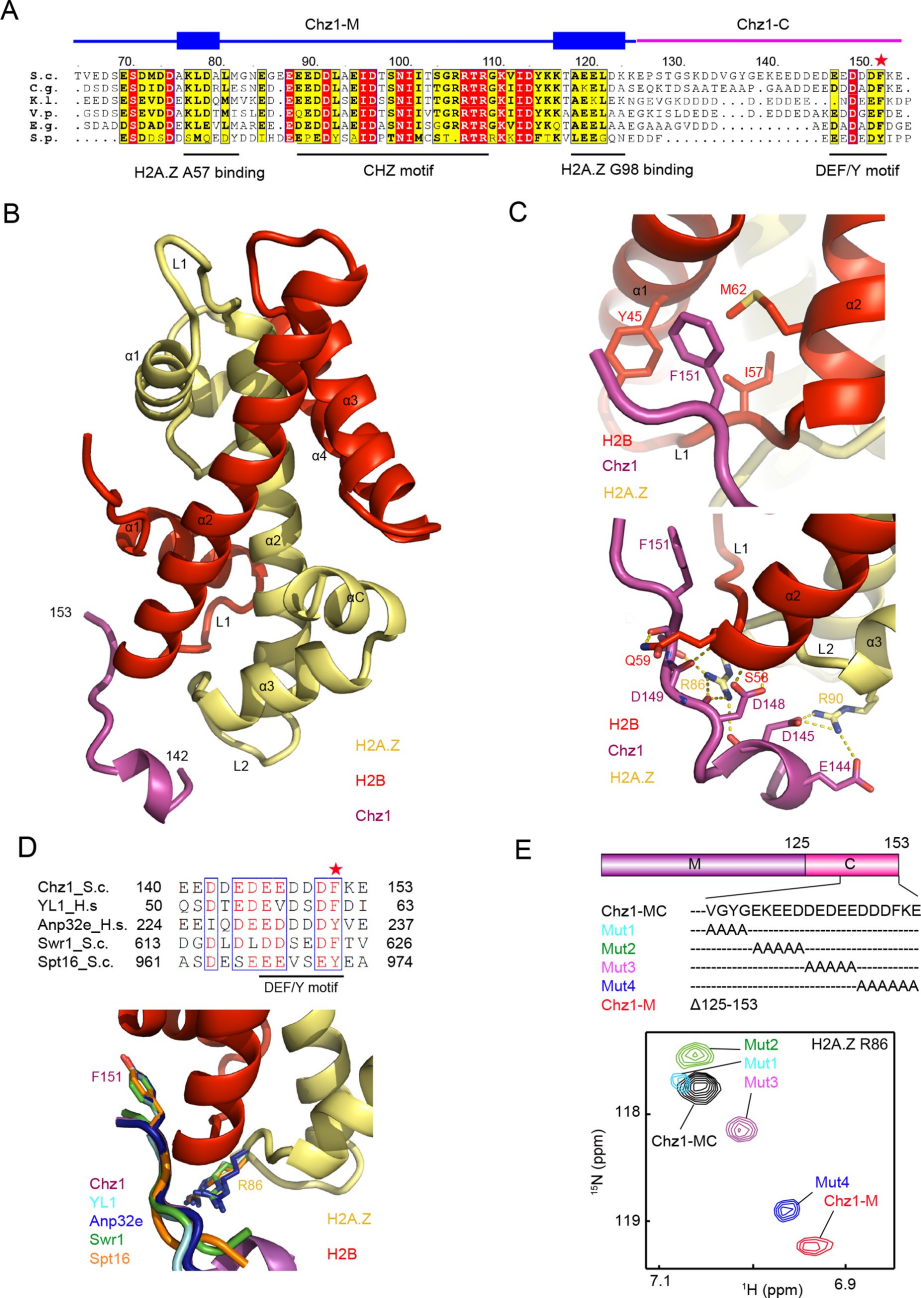
Structure and interaction of Chz1-C in complex of H2A.Z-H2B. (A) Schematic view of aligned fungi Chz1-MC sequences. Blue and purple lines represent Chz1-M and Chz1-C. Black lines: CHZ motifs. The conserved Phe/Tyr residues are highlighted by star. (B) The overall structure of Chz1-C in complex with yeast H2A.Z-H2B. Magenta, Chz1-C; yellow, H2A.Z; red, H2B. (C) Close view of the interaction between Chz1 residue F151 and H2B pocket formed by the H2B α1 helix, L1 loop, and α2 helix (top panel) and interaction between Chz1-C polar residues and H2A.Z L2 loop and H2B L1 loop (lower panel). (D) Comparison of DEF/Y motif adopted by different histone chaperones. (Top) Aligned DEF/Y motif sequences with known structures. The Phe/Tyr residues are highlighted by star. (Bottom) Structural comparison of histone H2B pockets interacting with DEF/Y motifs from human YL1 (cyan), Anp32e (blue), Swr1 (green), Spt16 (orange). The F/Y residues corresponding to Chz1 F151, the histone H2A.Z R86 (and the H2A counterparts) serving as the arginine finger are highlighted in the structures. (E) Effect of Chz1 mutations on H2A.Z-H2B interaction revealed by CSP analysis. The ^1^H-^15^N HSQC spectrum of ^15^N labeled scZB in complex with Chz1-MC and mutated Chz1-MC (Mut1, Mut2, Mut3, Mut4) or Chz1-M are compared. The chemical shift of H2A.Z residue R86 in association with different Chz1 mutants are displayed in the same color as shown in the sequences of corresponding Chz1. The underlying data can be found in S2 Data. Anp32e, acidic leucine-rich nuclear phosphoprotein 32 family member e; CHZ, a defined Chz1 region showing sequence conservation across all species; Chz1, chaperone for H2A.Z-H2B; Chz1-C, C-terminal region of Chz1; Chz1-M, middle region of Chz1; Chz1-MC, middle and C-terminal region of Chz1; *C*.*g*., *Candida glabrata;* CSP, chemical shift perturbation; *E*.*g*., *Eremothecium gossypii*; DEF/Y, the consecutive D/E residues followed by a single aromatic residue; *H*.*s*., *Homo sapiens;* HSQC, heteronuclear single-quantum coherence; *S*.*c*., *Saccharmyces cerevisiae*; scZB, single-chain H2A.Z-H2B; *S*.*p*., *Schizosaccharomyces pombe*; Spt16, Suppressor of Ty 16; Swc2, SWR complex protein 2; Swr1, Swi2/snif2-related 1; YL1, Swc2 homolog in higher eukaryotes; *V*.*p*., *Vanderwaltozyma polyspora*.

To reveal the structural basis for recognition of H2A.Z-H2B dimer by Chz1-FL, we reconstituted Chz1 complexes by mixing recombinant Chz1 containing residues 64–153 (Fig 2A) with yeast scZB at 1:1 molar ratio and determined the crystal structure of Chz1-MC in the complex of scZB at a 1.65-Å resolution (Fig 2B, Table 2). The structure was solved by molecular replacement using the structure of H2A.Z-H2B dimer in the yeast nucleosome (protein data bank [PDB]: IF66) [20] and refined to work reliability factor (R_work_) of 18% and free reliability factor (R_free_) of 22% (Table 2). The final model contains Chz1 residues 142–153 and is missing residues 64–141 in Chz1-MC, likely due to the partial degradation of Chz1-MC protein (S2 Fig). Consistent with the aforementioned CSP mapping results, the complex structure showed that Chz1-C interacts with the H2B L1 loop and α1 and α2 helices, as well as with the H2A.Z L2 loop (Fig 2B, S2 Fig). The binding of scZB and Chz1-C, which buries a total area of 494 Å^2^ (calculated by protein data bank in europe, proteins interfaces structures and assemblies [PDBePISA] program), is stabilized by both hydrophobic and polar interactions. The side chain of Chz1 residue F151 inserts into the shallow hydrophobic pocket surrounded by Y45, I57 in H2B α1 helix and M62 in H2B α2 helix (Fig 2C). In addition, Chz1 residues E144, D145, D148, D149, and D150 form salt bridges and hydrogen bonds with R86 and R90 of H2A.Z as well as S58 and Q59 of H2B (Fig 2C). Because equivalent arginine residues are also present in H2A (i.e., R79 and R83), this interaction is not sufficient to determine H2A.Z specificity (but see below). This histone binding motif contributed by Chz1 engages the Z-B dimer in a conformation reminiscent of other ancestrally unrelated Z-B and A-B dimer binding proteins, namely, Swr1, Swc2, Anp32e, and suppressor of Ty 16 (Spt16; Fig 2D), and is expected to exclude nucleosomal DNA [10–12,21]. We refer to this common histone binding site as the DEF/Y motif hereafter, reflecting the consecutive D/E residues followed by a single aromatic residue (Fig 2A).

**Table 2:** Chz1-MC/H2A.Z-H2B data collection and refinement statistics.

**Data collection**	
Space group	*P1*
Cell dimensions	
*a*, *b*, *c* (Å)	42.08, 46.21, 56.66
α, β, γ (°)	72.4, 70.9, 70.6
Resolution (Å)	50.00–1.65 (1.71–1.65)
*R*_merge_	0.049 (0.655)
*I*/σ*I*	20.0 (1.7)
Completeness (%)	96.5 (93.5)
Redundancy	3.9 (3.7)
**Refinement statistics**	
Resolution (Å)	50.00–1.65
Number of reflections	40,827
*R*_work_ / *R*_free_	0.18 / 0.22
Number of. atoms	3,202
Protein	3,039
Ligand/ion	46
Water	117
Average B-factors	24.6
R.m.s. deviations	
Bond lengths (Å)	0.019
Bond angles (°)	1.88
Ramachandran favored (%)	98
Ramachandran allowed (%)	2
Ramachandran outliers (%)	0

Values in parentheses are for highest-resolution shell.

**Abbreviations:** Chz1, chaperone for H2A.Z-H2B; Chz1-MC, middle and C-terminal region of Chz1; Rfree, free reliability factor; Rwork, work reliability factor

To further dissect the determinant of the DEF/Y motif of Chz1 for binding Z-B, we performed alanine scanning analysis in and around the DEF/Y motif using the Chz1-MC fragment (Mutant Mut1–Mut4; Fig 2E). Using the chemical shift of R86 of H2A.Z as a readout for DEF/Y interaction, we compared the ^1^H–^15^N HSQC spectra of scZB-Chz1 complexes containing wild-type (WT) and mutated Chz1, examining the CSP changes of scZB residues. The substantial H2A.Z CSP changes observed in Chz1-M complex is likely due to the loss of binding, which is revealed by the ITC experiments (Fig 1B). In comparison with Chz1-MC WT, Mut1 (with alanine substitutions of VGYG), Mut2 (with alanine substitutions of EKEED), and Mut3 (with alanine substitutions of DEDEE) resulted in modest changes for CSP of H2A.Z R86. In contrast, Chz1-MC Mut4 (with alanine substitutions of DDDFKE) generated a drastic change for CSP of H2A.Z R86, thus suggesting that the last 6 residues of Chz1 play an essential role in Chz1-C recognition (Fig 2E). Moreover, comparison of the CSP profiles of Chz1-MC and Chz1-F151 reveals that F151A of the DEF/Y motif is required for interaction with the Η2Α.Ζ L2 and Η2Β L1 and α1 regions, because F151A ablated the interaction of Chz1-MC at these Z-B dimer sites (Fig 1D). We further mutated H2B Y45 and M62, which make up part of the hydrophobic pocket for Chz1 F151, to alanine and performed ITC analysis. In accordance with the structure, the H2B Y45A/M62A mutant exhibited an approximately 9-fold decrease of affinity (*Kd* of 1.1 μM) in comparison to WT H2B (*Kd* of 0.12 μM), underscoring the role of the aromatic residue in the DEF/Y motif in histone dimer binding (S3 Fig, Table 1). Taken together, we conclude that the DEF/Y motif of Chz1-C directly engages the arginine residues in H2A.Z L2 loop and a hydrophobic pocket in H2B for Z-B dimer interaction.

### Key residues confer H2A.Z preference

**Fig 3:**
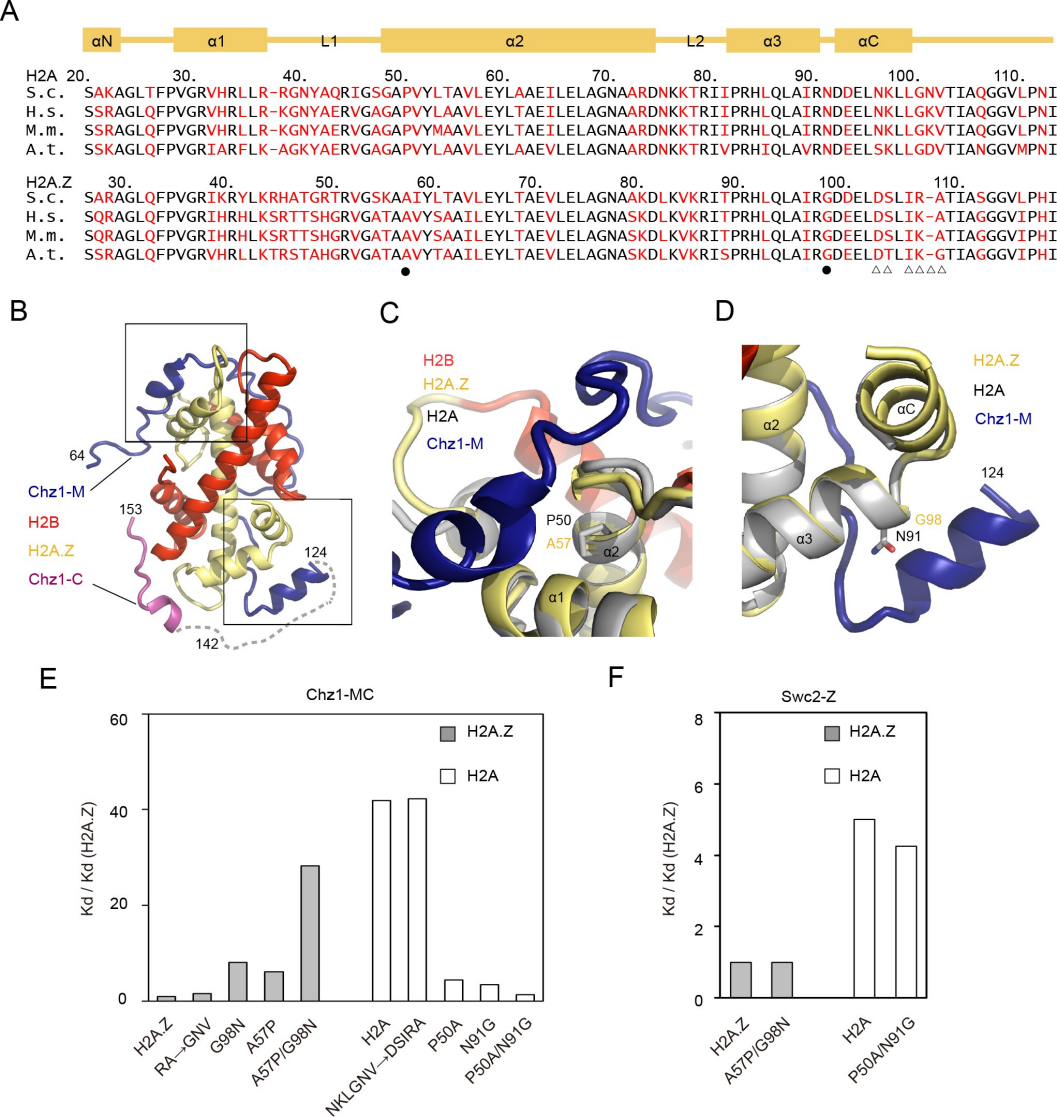
H2A.Z residues with key roles for preferential recognition of Chz1. (A) Schematic view of aligned H2A.Z and H2A. Shown on the top is a schematic view of the secondary structure of both histones. H2A.Z-specific residues and their H2A counterparts are colored in red. Residues interchanged for ITC analysis are highlighted with dots or triangles. Black dots highlight H2A.Z residues playing central roles in preferential recognition of Chz1. (B) Structural model of Chz1 in complex with H2A.Z-H2B. Structure of Chz1-C complex is superimposed with the structure of Chz1-M complex (PDB 2JSS). The dotted line stands for Chz1 residues 125–141, which are not visible in both structures. Blue: Chz1-M; magenta: Chz1-C; red: H2B; yellow: H2A.Z. (C) Close view of the binding interface between H2A.Z α2-helix and Chz1 αN-helix. H2A.Z residue A57 and its H2A counterpart P50 are colored in yellow and gray. H2A-H2B structure from nucleosome (PDB: 1ID3) is superimposed for comparison. (D) Close view of the binding interface between H2A.Z α3-helix and Chz1 αC-helix. H2A.Z residue G98 and its H2A counterpart N91 are colored in yellow and gray. H2A-H2B structure from nucleosome (PDB: 1ID3) is superimposed for comparison. (E) Effect of H2A.Z-H2A and H2A-H2A.Z interchanges on Chz1 recognition by ITC analysis. The underlying data can be found in S1 Data. (F) ITC analysis showing the effect of H2A.Z mutant A57G/G98N and H2A residues P50G/N91G on recognition of H2A.Z-chaperone Swc2. The underlying data can be found in S1 Data. *A*.*t*., *Arabidopsis thaliana*; Chz1, chaperone for H2A.Z-H2B; Chz1-C, C-terminal region of Chz1; Chz1-M, middle region of Chz1; *H*.*s*., *Homo sapiens*; ITC, isothermal titration calorimetry; *M*.*m*., *Mus musculus*; PDB, protein data bank; *S*.*c*., *Saccharmyces cerevisiae*; Swc2, SWR complex protein 2.

In light of the crystal structure and the NMR structure reported previously, we built a structural model of Chz1-FL in complex with the H2A.Z-H2B dimer, in which Chz1 extensively interacts with and wraps around the Z-B dimer (Fig 3A–3D). Chz1 residues 126–141, which are not observable in both structures, likely serve as a linker between Chz1-M and Chz1-C (Figs 2A and 3B). To elucidate the mechanism by which Chz1 preferentially recognizes H2A.Z, we interchanged H2A and H2A.Z residues displaying sequence specificity and measured the binding affinity of Chz1-MC mutants by ITC (Fig 3E). As previous studies showed that the lack of glycine in the αC-helix of H2A.Z is critical for H2A.Z chaperone recognition, we first interchanged H2A.Z residues R107/A108 and H2A residues N96/K97/L99/G100/N101/V102 with their counterpart residues to generate H2A.Z RA→GNV and H2A NKLGNV→DSIRA mutants for ITC analysis (Fig 3A). These mutations show no effect on Chz1-MC bindings (Fig 3E, Table 1 row 8 and 13), suggesting the lack of glycine in αC-helix may not dictate the H2A.Z preference (Fig 3A). Further structural analysis indicates that H2A.Z-specific residues A57 and G98 that are presented in the Chz1-M region may affect Chz1-MC interaction. On the one hand, H2A.Z residue A57 in the α2-helix engages with Chz1 residues 76–81 that form an α-helical structure (Figs 2A and 3C). Substitution of A57 with its H2A counterpart P50 might shift the H2A.Z L1 loop or H2A.Z α1 helix, causing structural changes that secondarily impair interactions between nearby surfaces of H2A.Z and Chz1 (Fig 3C). On the other hand, H2A.Z residue G98 locates right on the binding interface formed by the H2A.Z α3-helix and Chz1 residues 118–123 embedded in the α-helical structure (Figs 2A and 3D). We speculated that substitution of G98 with H2A residues N91 with a large side chain may disrupt the binding between Chz1 and H2A.Z α3-helix (Fig 3D). In accordance with the speculation, binding of Chz1-MC and H2A.Z mutants A57P or G98N display an approximately 6-fold (*Kd* of 0.74 μM) or approximately 8-fold (*Kd* of 0.97 μM) decrease of affinity compared with WT H2A.Z (Fig 3E). Moreover, H2A.Z double mutation A57P/G98N further decreases the binding by a factor of approximately 29 (*Kd* of 3.4 μM; Fig 3E). These results are in good agreement with the aforementioned structural analysis. Moreover, we observed the approximately 9.5-fold and approximately 12-fold higher Chz1-MC binding affinity for H2A mutants P50A (*Kd* of 0.53 μM) and H2A N91G (*Kd* of 0.42 μM), and the approximately 32-fold higher binding affinity for H2A double mutant P50A/N91G (*Kd* of 0.16 μM) than for WT H2A (Fig 3E). These results demonstrate the combined effect of H2A.Z residues G98 and A57 on Chz1 recognition (Fig 3E, Table 1). We conclude that H2A.Z-specific residues G98 and A57 are necessary and sufficient to confer H2A.Z the preferential recognition.

To investigate whether the recognition of H2A.Z G98 and A57 is exclusively found in Chz1 but not in other H2A.Z chaperones, we measured the binding of H2A.Z A57P/G98N and H2A P50A/N91G mutants to Swc2-Z, the 1–136 segment of the Swc2 subunit of the SWR1 complex [12]. Swc2-Z recognizes H2A.Z residues T88 and D103 and the lack of a glycine in the αC helix of H2A.Z. ITC results showed that the substitution of H2A.Z residues G98 and A57 with their H2A counterparts, or vice versa, fail to change the binding of Swc2-Z (Fig 3F). Taken together, our findings reveal a new mechanism by which Chz1-MC recognizes H2A.Z residues G98 and A57 for H2A.Z selection and provide the first structural insight into function of the highly conserved H2A.Z residues G98 and A57.

A previous study identified a number of fungal homologs of Chz1 and a family of higher eukaryotic proteins possessing significant sequence similarity to the CHZ motif [17]. Multisequence alignment revealed the presence of a CHZ motif in higher eukaryotic proteins, including *Arabidopsis thaliana* Chz1 (AtChz1) and mammalian HIRA-interacting protein 3 (HIRIP3) (S4 Fig). However, neither AtChz1 nor HIRIP3 harbors the H2A.Z G98/A57-binding domains and the DEF/Y motif that are critical for binding of Z-B dimer, suggesting their ability to interact with Z-B dimer might be altered (S4 Fig). We performed ITC to investigate the binding between AtChz1b and *A*. *thaliana* Z-B dimer and A-B dimer. Our results showed that the binding of AtChz1b and Z-B dimer decreases approximately 400-fold when NaCl concentration increases from 0.1 M NaCl (*Kd* = 0.05 μM) to 0.25 M NaCl (*Kd* = 19.9 μM; S4 Fig). It is worth noting that the salt dependence is also observed for yeast Chz1-M. The binding between Chz1-M and Z-B in 0.2 M NaCl yields a *Kd* of 0.02 μM, which is approximately 46-fold higher than the *Kd* (1.06 μM) measured in 0.5 M NaCl (Table 1, row 15 and 21; S4 Fig). Moreover, AtChz1b displays less preference for Z-B dimer over A-B dimer under both conditions (S4 Fig). We conclude that the higher eukaryotic proteins containing the CHZ motif are able to interact with both Z-B and A-B dimers, albeit with substantially reduced binding affinity and diminished H2A.Z preference. Conversely, fungal Chz1 proteins appear to have acquired preference for the H2A.Z variant through the aforementioned specificity elements flanking the conserved CHZ motif in concert with the C-terminal DEF/Y motif.

### Chz1 facilitates H2A.Z exchange and H2A.Z incorporation

**Fig 4:**
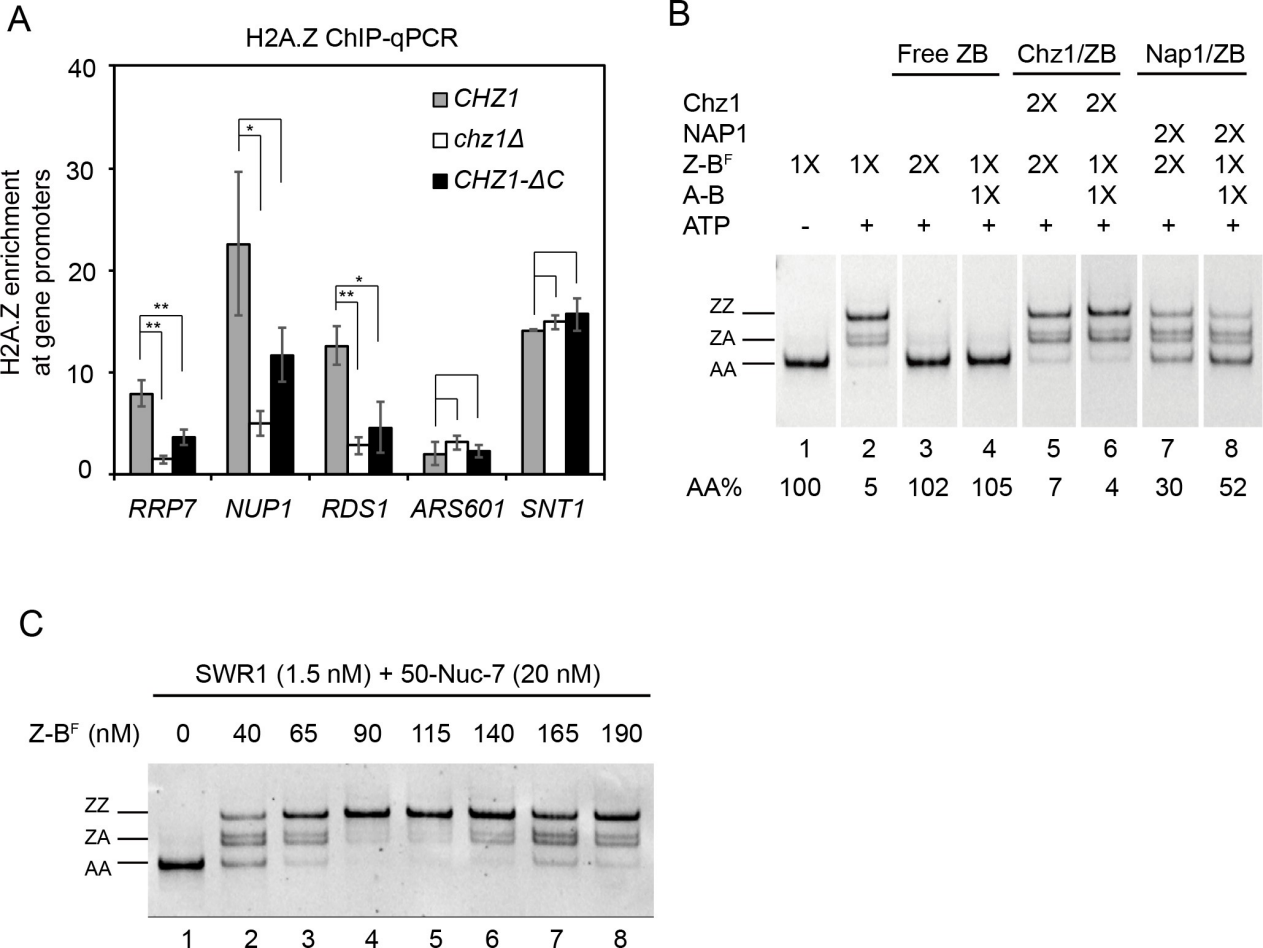
Effect of Chz1 on exchange and chromatin incorporation of H2A.Z. (A) ChIP-qPCR for H2A.Z-HA occupancy at the +1 nucleosome in yeast strains *CHZ1*, *chz1Δ*, and *CHZ1-ΔC*. Error bars, SD from 3 technical replicates. **p < 0.01. *p < 0.05. The underlying data can be found in S3 Data. (B) SWR1-mediated histone exchange in the presence or absence of excess histone dimer and Chz1/Nap1. The initial substrate concentration of H2A.Z-H2B dimer (115 nM) used for regular exchange assay was defined as 1×. Accordingly, 1×H2A-H2B dimer, 1×Chz1, and 1×Nap1 refer to concentrations in equal molar ratio of H2A.Z-H2B dimer. The order of loading samples is changed for better presentation. Z-B^F^ refers to Z-B dimer in which the H2B contains a FLAG tag. See S6 Fig for original gel. (C) Effect of excess H2A.Z-H2B dimer on H2A.Z exchange. Nucleosome is reconstituted on DNA with 146-bp widom 601 sequence flanking with 50-bp and 7-bp linker DNA at each end. AA, nucleosome containing 2 copies of H2A; Ars601, autonomously replicating sequence 601; ChIP-qPCR, chromatin immunoprecipitation and quantitative PCR; Chz1, chaperone for H2A.Z-H2B; Nap1, Nucleosome assembly protein 1; Nup1, nuclear Pore 1; Rds1, regulator of drug sensitivity 1; Rrp7, ribosomal RNA processing 7; Snt1, SANT 1; ZA, nucleosome containing 1 copy of H2A and H2A.Z each; ZZ, nucleosome containing 2 copies of H2A.Z.

There are conflicting reports on whether Chz1 is required for H2A.Z deposition in vivo [15,17]. To re-examine the role of Chz1 in H2A.Z deposition in vivo and the functional significance of the Chz1-C domain, we performed chromatin immunoprecipitation and quantitative PCR (ChIP-qPCR) on WT, *chz1*Δ, or *CHZ1-C* deletion (*CHZ1-ΔC)* cells that express a triple hemagglutinin (3×HA)-tagged H2A.Z (Fig 4A, Tables 3 and 4). In *chz1Δ*, H2A.Z occupancy decreases relative to WT at the promoters of *RRP7*, *NUP1*, and *RDS1* (*RRP7*, 1.47 ± 0.34/7.88 ± 1.26, *p* < 0.01; *NUP1*, 5.01 ± 1.17/22.58 ± 7.07, *p* < 0.05; *RDS1*, 2.80 ± 0.81/12.57 ± 1.92, *p* < 0.01; *n*  = 3; Fig 4A) but remains largely unchanged at the *SNT1* promoter and the *ARS601* locus (*SNT1*, 14.93 ± 0.66/14.09 ± 0.04; *ARS601*, 3.11 ± 0.73/2.01 ± 1.12; *n*  = 3; Fig 4A). *CHZ1-ΔC* also exhibits a reproducible, but smaller, decrease in H2A.Z occupancy at *RRP7* and *RDS1* (*RRP7*, 3.62 ± 0.73/7.88 ± 1.26, *p* < 0.01; *RDS1*, 4.58 ± 2.48/12.57 ± 1.92, *p* < 0.05; *n*  = 3; Fig 4A). We conclude that Chz1 is required for optimal H2A.Z deposition in a subset of genes and that the Chz1-C domain might affect these specific Chz1-mediated depositions in vivo.

**Table 3:** Primers used for the ChIP-qPCR experiments.

Primer name	Primer sequence (5’–3’)
*NUP1-F*	AACATGCAGTGTGTCTTCAACATCTGATTCC
*NUP1-R*	AAATAAGTGATGAGGAACTCAATAAGGAC
*RRP7-F*	GAAATGCAACGAAGCTTCCTGGC
*RRP7-R*	TGAGAGGATGGTAAAGCAAGAGGC
*RDS1-F*	GACCCGTGCAGATCACTATTACA
*RDS1-R*	GCAGTTTATCACATTTCCGTTTG
*ARS601-F*	GCGTAACAAAGCCATAATGCCTCC
*ARS601-R*	CTCGTTAGGATCACGTTCGAATCC
*SNT1-F*	AGTTAGTAGGCAAATTATTAAGCAGGCCC
*SNT1-R*	CGCCTATGGAAAACGGCAAAAGGG

**Abbreviations:** Ars601, autonomously replicating sequence 601; ChIP-qPCR, chromatin immunoprecipitation and quantitative PCR; F, forward; Nup1, nuclear Pore 1; R, reverse Rds1, regulator of drug sensitivity 1; Rrp7, ribosomal RNA processing 7; Snt1, SANT 1

**Table 4:** Strains for ChIP-qPCR of the yeast cells used in this study.

*CHZ1 WT or mutant*	Yeast strain [plasmid ]
*CHZ1*	MATa his3Δ1 leu2Δ0 met15Δ0 ura3Δ0 Swr1::3FLAG Htz1-3HA-HIS3MX6 Chz1::HPH [pRS316-Chz1-WT].
*chz1Δ*	MATa his3Δ1 leu2Δ0 met15Δ0 ura3Δ0 Swr1::3FLAG Htz1-3HA-HIS3MX6 Chz1::HPH [pRS316]
*CHZ1-ΔC*	MATa his3Δ1 leu2Δ0 met15Δ0 ura3Δ0 Swr1::3FLAG Htz1-3HA-HIS3MX6 Chz1::HPH [pRS316-Chz1-ΔC]

**Abbreviation:** ChIP-qPCR, chromatin immunoprecipitation and quantitative PCR; Chz1, chaperone for H2A.Z-H2B; SWR1, Swi2/snif2-related 1

We next performed a histone exchange assay to investigate whether the addition of Chz1 to the Z-B dimer influences SWR1-mediated H2A.Z replacement (Fig 4B). Similar to results of a previous study in which an excess of A-B dimer inhibited H2A.Z exchange in vitro [22], titration with Z-B dimer showed that H2A.Z exchange is also inhibited by excess Z-B (Fig 4C). An equimolar level of Chz1 efficiently alleviates inhibition by Z-B dimer and A-B dimer (lane 5, 6), whereas equimolar Nap1 only partially alleviates these inhibitions (lane 7, 8; Fig 4B). These results suggest that Chz1 facilitates H2A.Z exchange by alleviating the inhibition of excess histone dimers. Moreover, for optimal facilitation, Z-B dimer association with Chz1 is more effective than with Nap1. As the majority of budding yeast Z-B dimer is associated with histone chaperone Chz1 and Nap1 [9], these results suggest that the partial redundancy of Chz1 and Nap1 might affect H2A.Z incorporation by modulating H2A.Z replacement.

### Chz1 exhibits histone chaperone activity

**Fig 5:**
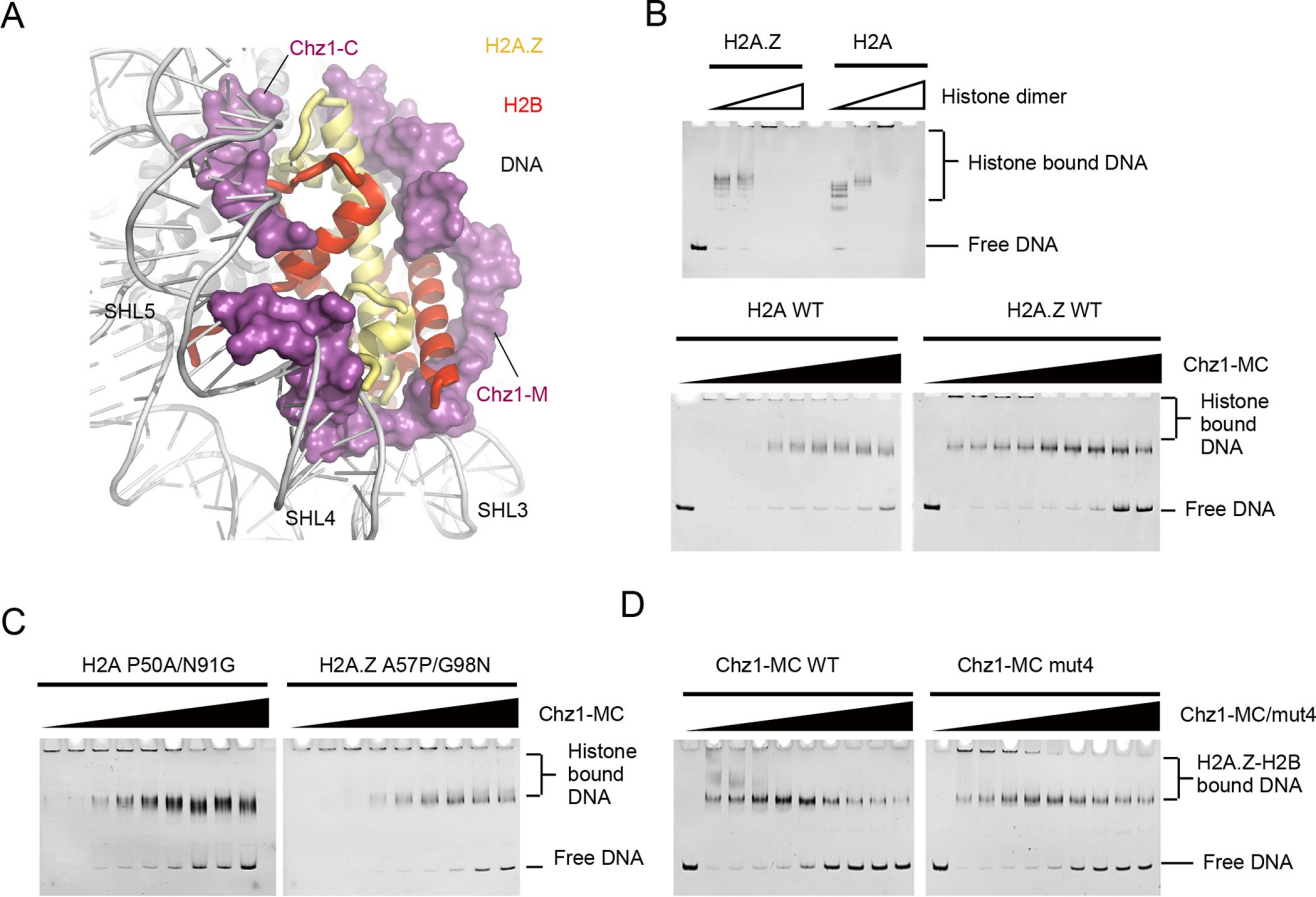
Effect of Chz1 and H2A.Z interaction on chaperone property. (A) Overlay of the Chz1-MC/H2A.Z-H2B complex structure with the H2A.Z nucleosome structure. Surface representation of Chz1-M and Chz1-C show structural incompatibility with nucleosomal DNA of SHL3/4 and SHL5, respectively. (B) (from the top): Effect of scZB and scAB on the formation of histone–DNA aggregations. Effect of Chz1-MC in dissolving scZB and scAB aggregations. (C) Effect of Chz1-MC in dissolving aggregation formed by H2A.Z A57P/G98N mutant and by H2A P50A/N91G mutant. (D) Effects of Chz1-MC Mut4 in dissolving scZB aggregations. Chz1, chaperone for H2A.Z-H2B; Chz1-C, C-terminal region of Chz1; Chz1-M, middle region of Chz1; Chz1-MC, middle region and C-terminal region of Chz1; scAB, single-chain H2A-H2B; scZB, single-chain H2A.Z-H2B; SHL, super helical location; WT, wild type.

One explanation for how Chz1 alleviates the inhibitory effect of excess Z-B dimers in the histone exchange reaction is that Chz1 functions to prevent H2A.Z from forming histone–DNA aggregates. Consistent with this view, structural superimposition of the Chz1-MC bound Z-B dimer and nucleosomal Z-B showed that Chz1-MC and nucleosomal DNA are mutually exclusive in 3D space (Fig 5A). Electrophoretic mobility shift assay (EMSA) shows that Chz1-MC dissolved histone–DNA aggregates formed by both Z-B and A-B dimers, albeit with higher preference for dissolving Z-B aggregates (Fig 5B). Further investigation of Chz1 mutations revealed that Chz1-MC dissolves aggregates formed by H2A mutant P50A/N91G more effectively than those formed by H2A.Z mutant A57P/G98N (Fig 5C). Moreover, WT Chz1-MC dissolved the Z-B dimer aggregates more effectively than Chz1-MC Mut4 (Fig 5D). These results are in accordance with the structure of the complex in which Chz1-MC shields the Z-B dimer from binding to DNA at super helical location (SHL) 3-SHL 5 and underscore the key roles of Chz1-M and Chz1-C in dictating the H2A.Z-specific chaperone activity of Chz1.

## Discussion

It has been over 10 years since the discovery of budding yeast Chz1, the first H2A.Z-specific histone chaperone. Although the structure of the Chz1-M in the complex of H2A.Z-H2B dimer has been previously determined, the mechanism by which Chz1 preferential recognizes H2A.Z remained unclear. Our present study elucidates the molecular basis for preferential recognition of the H2A.Z-H2B dimer and demonstrates that Chz1-M and Chz1-C regions function cooperatively to dictate the preference for H2A.Z.

The mechanism by which Chz1 recognizes H2A.Z residues A57 and G98 for specificity is distinct from those of known H2A.Z chaperones (S5 Fig). Although previous studies have shown that H2A.Z G98 mediates YL1-mediated H2A.Z recognition [11,12], the identification of H2A.Z residue A57 provides the first insight into the critical role of a highly conserved H2A.Z-specific residue in the α2 helix of the histone fold. Sequence comparison of H2A.Z within the histone-fold domain reveals that 43 residues out of 94 are distinct from H2A and highly conserved within the H2A.Z family (S5 Fig). Genetic studies have revealed the functional importance of conserved H2A.Z sequences throughout the histone fold and N-terminal region, but the structural basis for conserved H2A.Z functions have been determined only for a few residues in the C-terminal half [11–13]. As mentioned above, several conserved H2A.Z residues in the C-terminal loop 2 and α3 helix engage with Z-B binding domains of YL1 [12], Swr1 [10], and Anp32e [13], but structural interactions for conserved residues in the N-terminal half of H2A.Z have been obscure. To our knowledge, our findings provide the first structural insight into the role of A57 in the α2 helix of H2A.Z. The inclusion of A57 with G98 as specificity determinants demonstrates the novel combinatorial nature of H2A.Z recognition by Chz1 and raises possibilities of combinatorial specificity functions for other conserved H2A.Z residues among the emerging repertoire of H2A.Z-interacting proteins. It is also noteworthy that H2A P50, the equivalent H2A.Z A57 residue, plays a critical role in nucleosomal H2A recognition by SWR1 and is critical for SWR1-catalyzed H2A.Z incorporation. Substitution of H2A P50 with the H2A.Z-specific alanine substantially reduced replacement of the nucleosomal A-B dimer by the Z-B dimer [23].

The DEF/Y motif, which is located at the C terminus of budding yeast Chz1 and its fungal homologs, is found in other evolutionarily unrelated chromatin remodeling factors. The binding mode of the DEF/Y motif in Chz1-C with the Z-B dimer resembles those adopted by the A-B chaperone Spt16 and the Z-B chaperones YL1, Swr1, and Anp32e (Fig 2D). Previous studies suggested that the DEF/Y motif alone does not contribute to Z-B preference given that the DEF/Y motif interacts with H2B residues and common H2A/H2A.Z residues. Nonetheless, the DEF/Y motif is required by the Chz1-MC fragment for optimal and specific recognition of H2A.Z. We demonstrated that the binding of the DEF/Y motif to Z-B indirectly enhances preferential recognition of the Z-B dimer. We speculate that by anchoring Chz1-C onto the arginine of H2A.Z and the hydrophobic pocket of H2B, the C-terminal end of Chz1-M is guided to recognize H2A.Z G98. The cooperative use of direct and indirect mechanisms for specific recognition of H2A.Z is novel and raises interesting possibilities for the structural basis of H2A.Z recognition by other interacting factors. In addition, the structure of the Chz1-C with Z-B dimer suggests that the DEF/Y motif caps the arginine finger R86 involved in DNA minor groove interaction. Thus, another function of DEF/Y motif is likely to prevent unregulated histone–DNA interactions, consistent with our biochemical observations.

A family of higher eukaryotic proteins, such as AtChz1 and mammalian HIRIP3, present significant sequence similarity to the CHZ motif. However, the sequence of AtChz1 and HIRIP3 show limited similarity to yeast Chz1 in regions outside the CHZ motif, and they lack the H2A.Z A57/G98 binding domain and the DEF/Y motif. Our study shows that AtChz1 is able to interact with both Z-B and A-B dimers, albeit with substantially reduced binding affinity and diminished H2A.Z preference (S4 Fig). The CHZ motif has been adopted by a large variety of proteins for histone H2A/H2A.Z-H2B dimer interactions, and it appears to be a common mechanism among all eukaryotes. Fungal Chz1 has extended that mechanism to include specificity for the Z-B dimer.

Proteins like YL1, Swr1, and Anp32e are designated as H2A.Z-specific chaperones because they show binding preference for Z-B dimer. All these proteins function as the structural components of remodeling complexes like SRCAP or P400. Disruption of their binding with Z-B dimer has substantial effects on H2A.Z incorporation or eviction. In contrast, Chz1 is not deployed as a component of a chromatin remodeling complex nor causes the remodeling defects when depleted from cells. Rather, Chz1 controls the supply of Z-B dimer substrate and functions as a bona fide H2A.Z-specific chaperone.

In cells, H2A.Z-H2B dimers are not free but almost always bound by histone chaperones, such as Chz1, Nap1, FACT, and Fk506-binding nuclear protein 3/4 (Fpr3/4), raising the question of why cells have evolved such a redundant histone chaperone system to sequester the free histones [17]. One reason is that free Z-B dimers are potentially toxic, because unregulated binding with DNA may lead to aggregation and interfere with nuclear processes, including SWR1’s remodeling function. The fact that Chz1 can dissociate non-nucleosomal H2A.Z-DNA complexes and alleviate the inhibitory effect of excess H2A.Z-H2B dimers on SWR1 is consistent with this idea. Although a large fraction of H2A.Z-H2B dimers are associated with Nap1 in WT cells, Nap1 is less efficient in assisting H2A.Z deposition than Chz1 [17]. In fact, too much Nap1 itself inhibits SWR1 activity (S6 Fig, lane 7 and lane 9). Further experiments will be required to understand the basis of Nap1’s inhibition and the potential interplay among the different H2A.Z chaperones in modulating the deposition of H2A.Z. In summary, through the structural interactions reported herein, Chz1 can function as a bona fide H2A.Z chaperone to facilitate SWR1-catalyzed H2A.Z deposition by relieving the inhibitory effects of excess free Z-B dimers.

## Materials and methods

### Protein expression and purification

The plasmids of scZB, scAB, and Chz1-M were used as in previous studies [19]. Briefly, the coding sequences of scZB and scAB were constructed into pET17b vector, and the coding sequences of Chz1-M were constructed into pET42b vector carrying a 6×His tag in its C terminus. The Chz1-FL and its related mutants were constructed into pET28a vector carrying a 6×His tag in its N terminus.

For the expression of scZB, scAB, Chz1, and their mutants, plasmids were transformed and grown in *Escherichia coli* BL21-CodonPLUS(DE3)-RIPL cells (Stratagene La Jolla, CA, USA) to an OD 600 of 0.7 and induced with 0.5 mM isopropyl b-D-thiogalactoside (IPTG) (Amresco, Solon, OH, USA) in LB media for 4 h at 37°C.

For the purification of Chz1 and its mutants, cells were resuspended in lysis buffer containing 20 mM Tris-Cl (pH 8.0), 500 mM NaCl, and 10 mM imidazole, disrupted by high-pressure cell disruptor, and centrifuged at 16,000 rpm for 45 min. The supernatant was loaded onto a column packed with Ni-NTA (Qiagen, Dusseldorf, Germany), incubated for 2 h, washed with lysis buffer, and eluted in the buffer containing 20 mM Tris-Cl (pH 8.0), 500 mM NaCl, and 250 mM imidazole. The eluate was dialyzed into 20 mM Tris-Cl (pH 8.0), 150 mM NaCl for overnight; loaded onto Hitrap Q (GE Healthcare, Little Chalfont, Buckinghamshire, UK) with buffer containing 20 mM Tris-Cl (pH 8.0), 150 mM NaCl and eluted running a linear gradient of 10 column volumes of elution buffer containing 20 mM Tris-Cl (pH 8.0), 1 M NaCl. Fractions were pooled and dialyzed against 20 mM MES (pH 6.0), 200 mM NaCl for future use.

For the purification of scZB, scAB, and their mutants, cells were resuspended in lysis buffer containing 20 mM Tris-Cl (pH 7.4), 500 mM NaCl, disrupted by high-pressure cell disruptor, and centrifuged at 16,000 rpm for 45 min. The supernatant was loaded onto a column packed with SP (Sigma, Saint Louis, MO, USA) incubated for 2 h, washed with lysis buffer, and eluted in the buffer containing 20 mM Tris-Cl (pH 7.4), 1 M NaCl. The eluate was dialyzed into 20 mM Tris-Cl (pH 8.0), 500 mM NaCl for overnigh; loaded onto Hitrap SP (GE Healthcare, Little Chalfont, Buckinghamshire, UK) with buffer A containing 20 mM Tris-Cl (pH 8.0), 500 mM NaCl; and eluted running a linear gradient of 10 column volumes of elution buffer containing 20 mM Tris-Cl (pH 7.4), 1 M NaCl. Fractions were pooled and dialyzed against 20 mM MES (pH 6.0), 200 mM NaCl for future use.

### Crystallization, data collection, processing, and structure determination

For crystallization of the complex of Chz1-MC and scZB, protein samples were mixed with 1:1 molar ratio and followed by gel filtration Superdex 200 column (GE Healthcare, Little Chalfont, Buckinghamshire, UK); after harvesting the peak of the complex, we concentrated the samples to 20 mg/ml for crystallization.

Crystallization of the complex was performed at 16°C using the hanging drop vapor diffusion method. The crystals were grown for about 1 month in 0.1 M Bicine (pH 9.0), 2% v/v 1,4-Dioxane, 10% Polyethylene glycol 20000 (Hampton Research, Aliso Viejo, CA, USA). X-ray diffraction data were collected at the Shanghai Synchrotron Radiation Facility (SSRF) on beamline BL17U. Data were processed, integrated, and scaled using the HKL2000 package [24]. The crystals belong to the *P1* space group with unit cell parameters a = 42.08 Å, b = 46.21 Å, c = 56.66 Å, and α = 72.37°, β = 70.92°, γ = 70.60° [25]. The structure was solved through the molecular replacement method [26]. The initial searching model was from the nucleosome core particle crystal structure containing the histone variant H2A.Z (PDB code 1F66). Manual model building and refinement were performed with COOT and PHENIX (https://www.phenix-online.org/) [27,28]. Structural figures were drawn with the program PyMOL (http://www.pymol.org/) [29]. The interface area was calculated by PDBePISA [30].

### ITC experiments

The ITC experiments were performed using a MicroCal VP-ITC at 25°C (Malvern, Malvern, United Kingdom). All the samples were dialyzed into the buffer of 20 mM MES (pH 6.0), 0.5 M NaCl before the experiments. The 500 μl of histone chaperones (e.g., 50 μM or 150 μM Chz1 and its mutants) or Swc2 was injected into the cells containing 1.4 ml histones (e.g., 5 μM scZB or 15 μM scAB and their mutants). A total of 29 injections (each of 10 μl) were made, the heat released was analyzed, and one site was processed using Origin8.

### NMR experiments

All the NMR experiments were performed on a Varian 600-MHz spectrometer (Varian, Midland, ON, Canada). Experiments of 2D ^1^H-^15^N HSQC were performed. NMRPipe and Sparky were used to process the spectra (http://www.cgl.ucsf.edu/home/sparky/).

### Chz1 chaperone property activity analysis

The chaperone assay was modified from what was described in the early study [12]. As a control, the 147 bp Widom 601 sequence DNA (0.5 μM) was mixed with scAB or scZB at concentrations of 2, 4, or 8 μM and was subsequently incubated in 20 mM MES (pH 6.0) and 0.5 M NaCl in a total volume of 20 μL. To investigate the effects of Chz1-MC or its mutants, histones (8 μM) were incubated with 0.5, 1, 2, 3, 4, 5, 6, 7, or 8 μM of Chz1-MC or its mutants in 20 mM MES (pH 6.0) and 0.5 M NaCl. Binding of Chz1-MC or its mutants to histones was allowed to proceed at 4°C for 15 min before the addition of DNA. In addition, a DNA-free control containing only Chz1-MC at the concentration corresponding to the highest titration point with DNA alone was also used. Histone mutants were performed using the same protocol; 60% (v/v) sucrose was added to the reaction system after the reactions, precipitates were removed by centrifugation, and the remaining soluble complexes were separated with native PAGE.

### ChIP-qPCR

ChIP assays were carried out as reported earlier [10]. Briefly, yeast cells were grown to mid-log phase in the CSM-URA medium (20 g/l glucose, 6.7 g/l YNB, and 0.77 g/l CSM-URA) with an OD 600 of 0.7–0.9. Cells were harvested and cross-linked by suspending in 1% formaldehyde and incubated for 15 min at room temperature. The reaction was quenched by adding glycine to a final concentration of 125 mM and sitting at room temperature for another 5 min. Cells were lysed with glass beads in lysis buffer (50 mM Na-HEPES [pH 7.5], 5 mM EDTA, 150 mM NaCl, 1% Triton X-100, 0.1% sodium deoxycholate, 2 mM PMSF) supplemented with Roche protease inhibitor tablets (Roche, Mannheim, Germany). Cells extracts were sonicated to shear the DNA to an average size of 0.5 kb using Focused-ultrasonicator (Covaris M220, Woburn, Massachusetts, USA). The cells were centrifugated at 16,000 rpm for 10 min at 4°C; the supernatant was collected and incubated with 5 μL anti-HA antibody (A2095-1ML, Sigma, Saint Louis, MO, USA) for overnight in a cold room with rotation in a total volume of 2ml, which was followed by incubation with protein G magnetic beads (Millipore, Billerica, MA, USA) for 3 h at 4°C. Protein-bound beads were separated by magnetism, and the beads were washed for 5 min sequentially with 1 ml lysis buffer, 1 ml lysis buffer added with 500 mM NaCl, 1 ml washing buffer (10 mM Tris-HCl [pH 8.0], 0.25 M LiCl, 0.5% NP-40, 0.5% sodium deoxycholate, 1 mM EDTA), and 1 ml of TE (pH 8.0). Protein-DNA complexes were eluted with TE buffer supplemented with 1% SDS and incubated at 65°C overnight to reverse cross-linking. Samples were further treated with proteinase K (0.1 mg/ml) at 37°C for 60 min. DNA was purified by phenol extraction and ethanol precipitation. Purified DNA was analyzed by running real-time PCR reactions on ABI 7300 (Applied Biosystems, Life Technologies, Foster City, CA USA) with FastStart Universal SYBR Green Master (Rox) Mix (Roche, Mannheim, Germany), and the data were collected and analyzed with ABI7300 system SDS software.

### Histone exchange assay

The histone exchange assay was conducted as previously described [22]. Each reaction is 25 μl. The concentrations of SWR1 and AA nucleosomes are 1.5 nM and 20 nM, respectively. The concentration of H2A.Z-H2B dimer showing an optimal exchange efficiency is 115 nM, which is defined as the 1×concentration. The ZB exchange is inhibited with the addition of 1× H2A.Z-H2B or 1× H2A-H2B. For the histone chaperone rescue experiments, Chz1 and Nap1 were added along with H2A-H2B or H2A.Z-H2B at the final concentrations equal to the sum total of AB and ZB dimers.

## Supporting information

S1 Fig Comparison of ^1^H–^15^N HSQC spectra of scZB in complex with Chz1-FL (black) and Chz1-M (red). The underlying data can be found in S2 Data. (a) The ^15^N -labeled scAB forms a 1:1 complex with nonlabeled Chz1-M or nonlabeled Chz1-FL, respectively. The ^1^H–^15^N backbone resonances of scZB in complex with Chz1-M are assigned in a previous study and served as a reference. Highlighted in the rectangles are scZB residues showing remarkable chemical shift changes in both HSQC spectra. (b) Close view of scZB residues, which are highlighted in rectangles. Chz1, chaperone for H2A.Z-H2B; Chz1-FL, full-length Chz1; Chz1-M, middle region of Chz1; HSQC, heteronuclear single-quantum coherence; scAB, single-chain H2A-H2B; scZB, single-chain H2A.Z-H2B.
(TIF)

S2 Fig **The unit cell that contains 2 molecules of Chz1-scZB complex (A) and the omit map for Chz1 residues 142–153 (B).** Highlighted in (B) are key H2B residues interacting with Chz1 residues 142–153. The omit map of Chz1 residues 142–153 is generated by PHENIX and contoured at the 1.0 σ level at 1.65 Å resolution. The figure was generated using Pymol. Chz1, chaperone for H2A.Z-H2B; scZB, single-chain H2A.Z-H2B.
(TIF)

S3 Fig Effect of H2B mutations on Chz1-C interaction revealed by ITC. The WT scZB and scZB with H2B Y45A/M62A mutation were titrated by Chz1-MC. The underlying data can be found in S1 Data. Chz1, chaperone for H2A.Z-H2B; Chz1-C, C-terminal region of Chz1; Chz1-MC, ITC, isothermal titration calorimetry; scZB, single-chain H2A.Z-H2B.
(TIF)

S4 Fig The CHZ motif–containing proteins in higher eukaryotes show less H2A.Z preference due to the absence of the H2A.Z G98/A57 binding domain and DEF/Y motif. (A) Schematic view of aligned Chz1-MC sequences from CHZ motif-containing eukaryotes. Blue and purple lines refer to Chz1-M and Chz1-C regions. Black lines refer to motif involve in histone dimer interaction. The Phe/Tyr residues are highlighted by stars. (B) ITC analysis of binding between AtChz1b (residues 253–463) and AtH2A.Z/AtH2B dimer in 0.1 M and 0.25 M NaCl. In contrast to yeast Chz1, AtChz1b displayed no preference for H2A.Z and showed a diminished binding in 0.25 M NaCl. The underlying data can be found in S1 Data. At, *Arabidopsis thaliana*; *C*.*g*., *Candida glabrata;* CHZ, a defined Chz1 region showing sequence conservation across all species; Chz1, chaperone for H2A.Z-H2B; Chz1-C, C-terminal region of Chz1; Chz1-M, middle region of Chz1; Chz1-MC middle and .C-terminal region of Chz1; *E*.*g*., *Eremothecium gossypii*; *K*.*l*., *Kluyveromyces lactis*; *S*.*p*., *Schizosaccharomyces pombe*; *S*.*c*., *Saccharmyces cerevisiae*; *V*.*p*., *Vanderwaltozyma polyspora*.
(TIF)

S5 Fig Comparison of H2A.Z-specifc residues conferring preference to H2A.Z chaperones. Schematic view of secondary structures of nucleosomal H2A (gray) and H2A.Z in complex of known H2A.Z chaperones (yellow). Extended H2A.Z αC-helix (orange). Residue differences between yeast H2A.Z and yeast H2A are in red; critical residues conferring H2A.Z preference to H2A.Z chaperones are highlighted in black dots. αC helix, alpha helix C terminal.
(TIF)

S6 Fig SWR1-mediated histone exchange in the presence or absence of excess histone dimer and Chz1/Nap1. The original gel of Fig 4C. Z-B^F^ refers to Z-B dimer in which the H2B contains a FLAG tag. Chz1, chaperone for H2A.Z-H2B; Nap1, nucleosome assembly protein 1; SWR1, Swi2/snif2-related 1; Z-B, H2A.Z-H2B.
(TIF)

S1 Data Excel file containing the numerical ITC data for Figs 1B and 1C, 3E and 3F, S3 and S4B. ITC, isothermal titration calorimetry. (XLSX)

S2 Data Excel file containing the numerical NMR data for Figs 1D, 2E, S1A and S1B. (XLSX)

S3 Data Excel file containing the numerical ChIP-qPCR data for Fig 4A. ChIP-qPCR, chromatin immunoprecipitation and quantitative PCR.
(XLSX)

## Abbreviations

αC: helix, alpha helix C terminal
A-B: H2A-H2B
Anp32e: acidic leucine-rich nuclear phosphoprotein 32 family member e
AtChz1: *Arabidopsis thaliana* Chz1
ChIP-qPCR: chromatin immunoprecipitation and quantitative PCR
Chz1: chaperone for H2A.Z-H2B
Chz1-C: C-terminal region of Chz1
Chz1-FL: full-length Chz1
Chz1-M: middle region of Chz1
Chz1-MC: middle and C-terminal region of Chz1
Chz1-N: N-terminal region of Chz1
Chz1-NM: N-terminal and middle region of Chz1
CSP: chemical shift perturbation
DEF/Y: the consecutive D/E residues followed by a single aromatic residue
EMSA: electrophoretic mobility shift assay
FACT: facilitates chromatin transcription
Fpr3: Fk506-binding nuclear protein 3
HIRIP3: HIRA-interacting protein 3
HSQC: heteronuclear single-quantum coherence
IPTG: isopropyl b-D-thiogalactoside
ITC: isothermal titration calorimetry
Nap1: nucleosome assembly protein 1
PDB: protein data bank
PDBePISA: protein data bank in europe, proteins interfaces structures and assemblies
p400: Protein 400
R_free_: free reliability factor
R_work_: work reliability factor
scAB: single-chain H2A-H2B
scZB: single-chain H2A.Z-H2B
SHL: super helical location
Spt16: suppressor of Ty 16
SRCAP: Snf2-related CBP activator protein
SSRF: Shanghai Synchrotron Radiation Facility
Swc2: SWR complex protein 2
SWR1: Swi2/snif2-related 1
Tip60: 60kDa Tat-interactive protein
WT: wild type
YL1: Swc2 homolog in higher eukaryotes
Z-B: H2A.Z-H2B
3×HA: triple hemagglutinin
