# Supplementary figures and images for: Structural insights into histone chaperone Chz1-mediated H2A.Z recognition and histone replacement
Source: PLoS Biol. 2019 May 20;17(5):e3000277. doi: 10.1371/journal.pbio.3000277 (PMC6544321; doi:10.1371/journal.pbio.3000277)

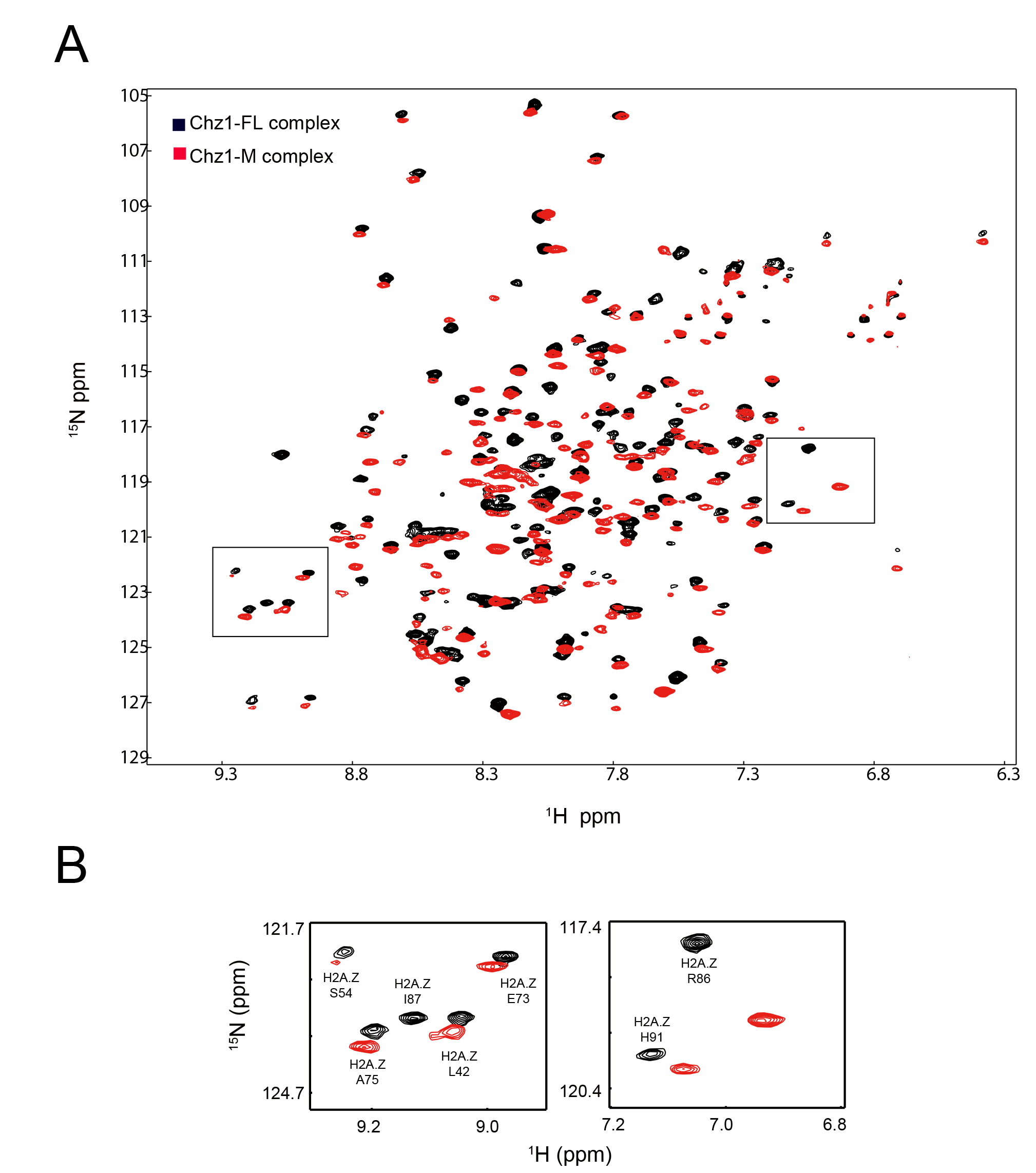

Supplement: S1 Fig — The underlying data can be found in S2 Data. (a) The 15N -labeled scAB forms a 1:1 complex with nonlabeled Chz1-M or nonlabeled Chz1-FL, respectively. The 1H–15N backbone resonances of scZB in complex with Chz1-M are assigned in a previous study and served as a reference. Highlighted in the rectangles are scZB residues showing remarkable chemical shift changes in both HSQC spectra. (b) Close view of scZB residues, which are highlighted in rectangles. Chz1, chaperone for H2A.Z-H2B; Chz1-FL, full-length Chz1; Chz1-M, middle region of Chz1; HSQC, heteronuclear single-quantum coherence; scAB, single-chain H2A-H2B; scZB, single-chain H2A.Z-H2B. (TIF) [file pbio.3000277.s001.tif]

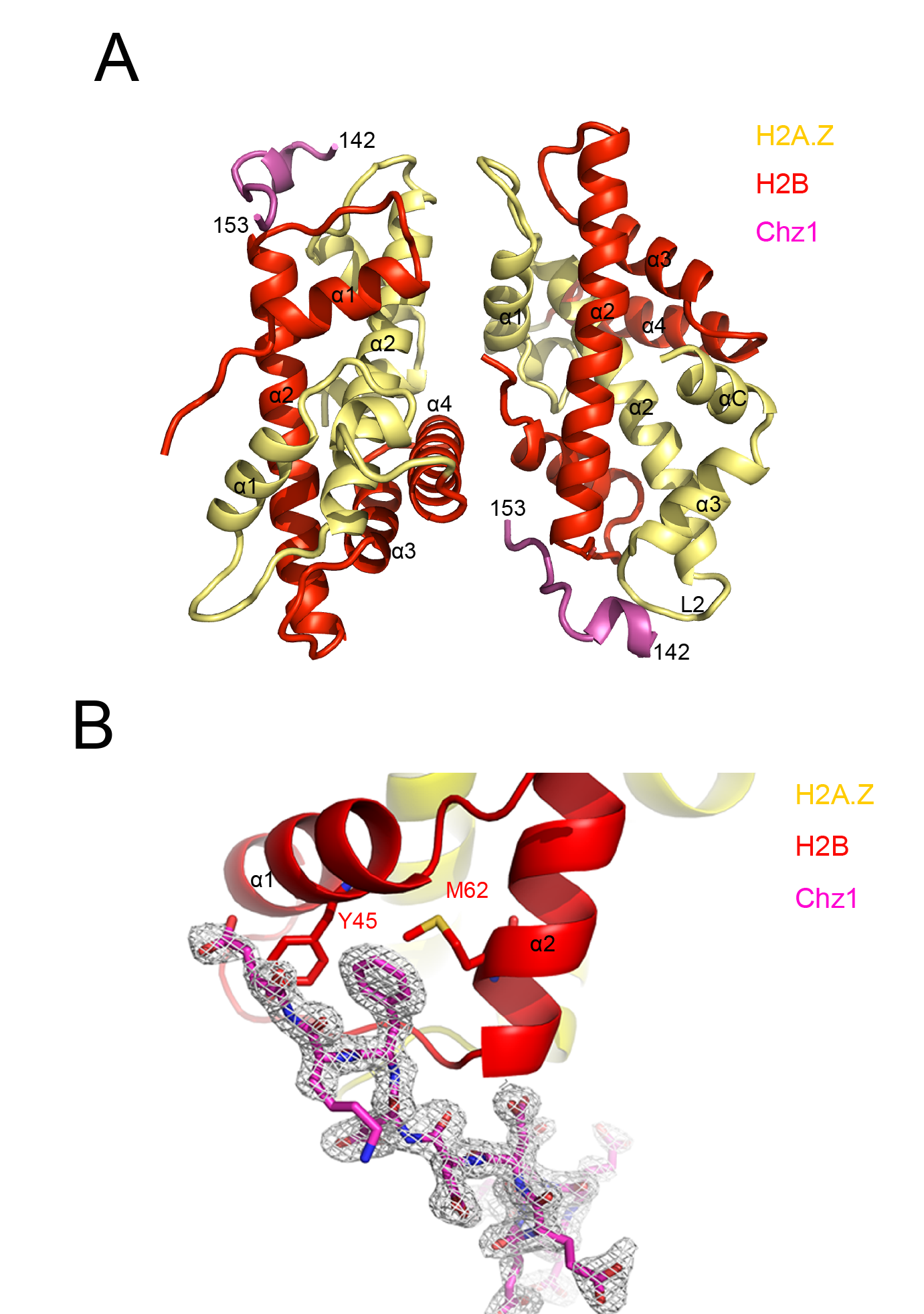

Supplement: S2 Fig — The unit cell that contains 2 molecules of Chz1-scZB complex (A) and the omit map for Chz1 residues 142–153 (B). Highlighted in (B) are key H2B residues interacting with Chz1 residues 142–153. The omit map of Chz1 residues 142–153 is generated by PHENIX and contoured at the 1.0 σ level at 1.65 Å resolution. The figure was generated using Pymol. Chz1, chaperone for H2A.Z-H2B; scZB, single-chain H2A.Z-H2B. (TIF) [file pbio.3000277.s002.tif]

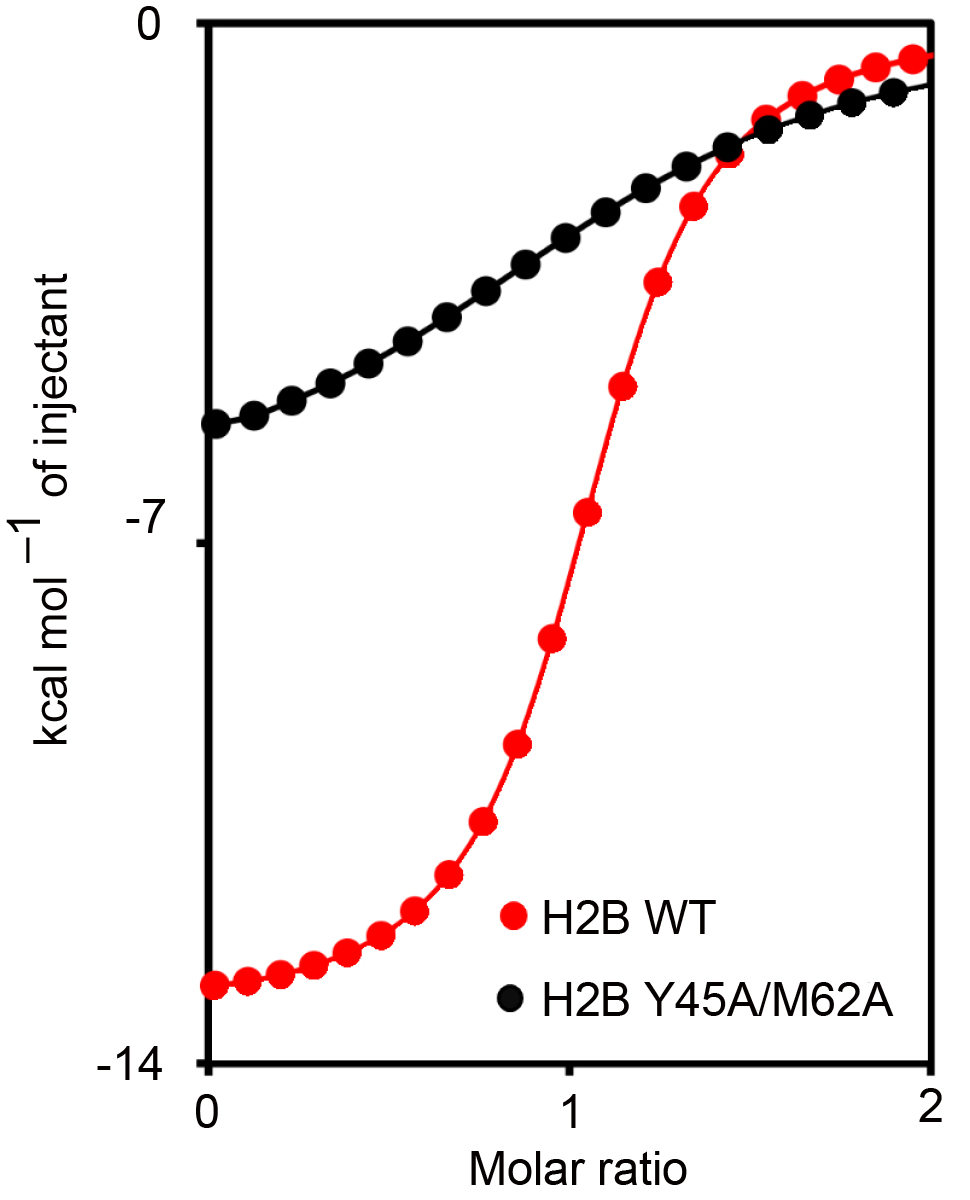

Supplement: S3 Fig — The WT scZB and scZB with H2B Y45A/M62A mutation were titrated by Chz1-MC. The underlying data can be found in S1 Data. Chz1, chaperone for H2A.Z-H2B; Chz1-C, C-terminal region of Chz1; Chz1-MC, ITC, isothermal titration calorimetry; scZB, single-chain H2A.Z-H2B. (TIF) [file pbio.3000277.s003.tif]

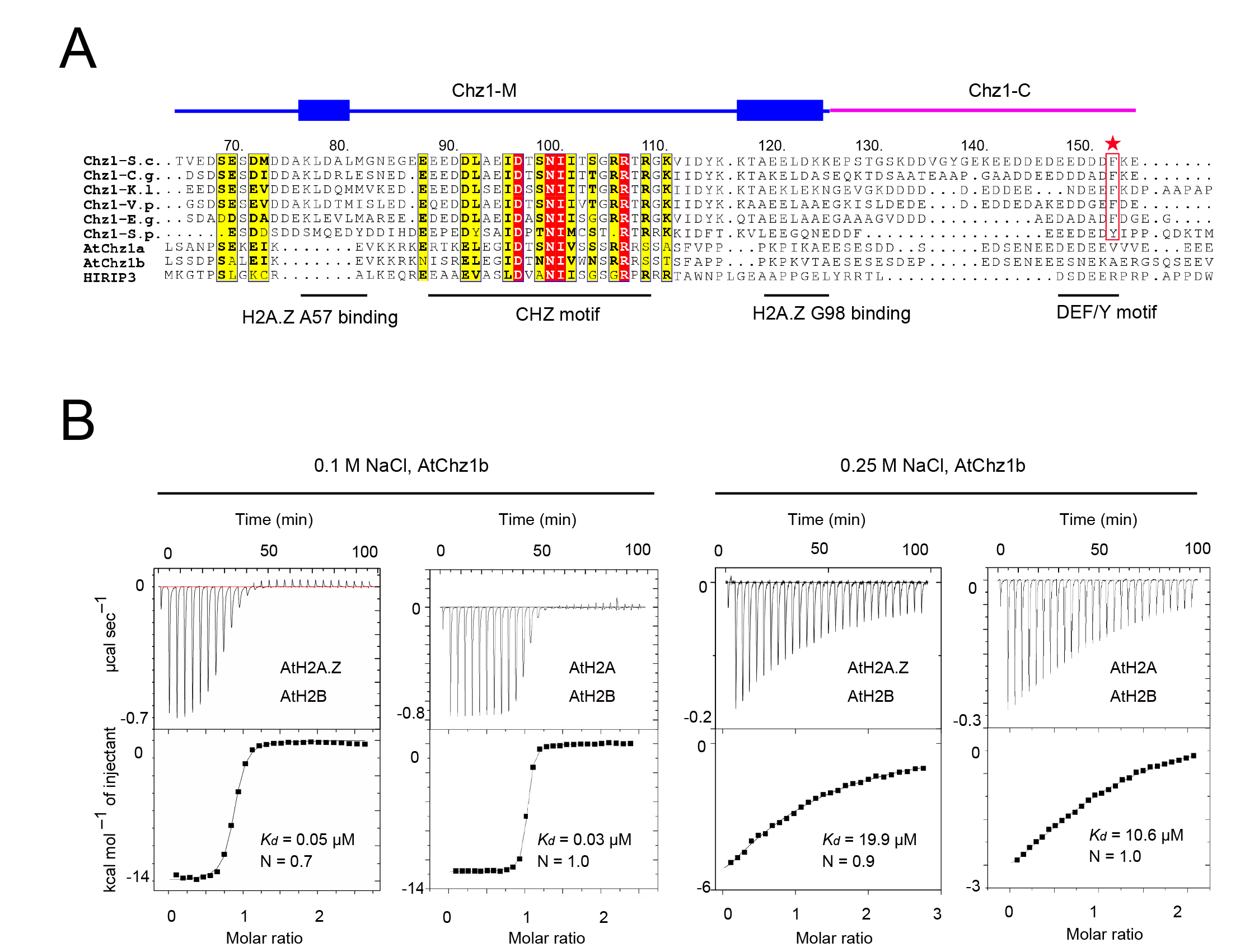

Supplement: S4 Fig — (A) Schematic view of aligned Chz1-MC sequences from CHZ motif-containing eukaryotes. Blue and purple lines refer to Chz1-M and Chz1-C regions. Black lines refer to motif involve in histone dimer interaction. The Phe/Tyr residues are highlighted by stars. (B) ITC analysis of binding between AtChz1b (residues 253–463) and AtH2A.Z/AtH2B dimer in 0.1 M and 0.25 M NaCl. In contrast to yeast Chz1, AtChz1b displayed no preference for H2A.Z and showed a diminished binding in 0.25 M NaCl. The underlying data can be found in S1 Data. At, Arabidopsis thaliana; C.g., Candida glabrata; CHZ, a defined Chz1 region showing sequence conservation across all species; Chz1, chaperone for H2A.Z-H2B; Chz1-C, C-terminal region of Chz1; Chz1-M, middle region of Chz1; Chz1-MC middle and .C-terminal region of Chz1; E.g., Eremothecium gossypii; K.l., Kluyveromyces lactis; S.p., Schizosaccharomyces pombe; S.c., Saccharmyces cerevisiae; V.p., Vanderwaltozyma polyspora. (TIF) [file pbio.3000277.s004.tif]

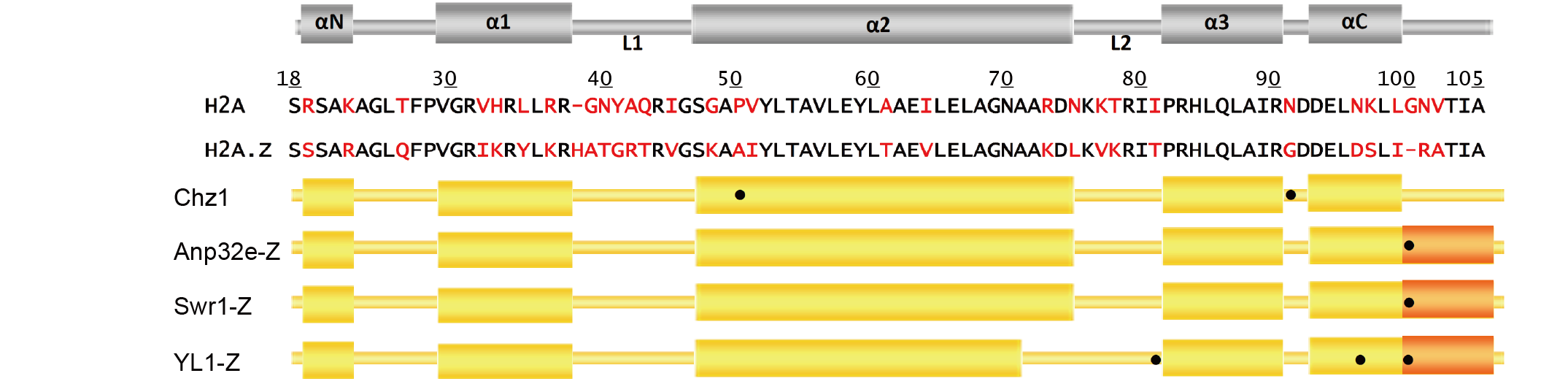

Supplement: S5 Fig — Schematic view of secondary structures of nucleosomal H2A (gray) and H2A.Z in complex of known H2A.Z chaperones (yellow). Extended H2A.Z αC-helix (orange). Residue differences between yeast H2A.Z and yeast H2A are in red; critical residues conferring H2A.Z preference to H2A.Z chaperones are highlighted in black dots. αC helix, alpha helix C terminal. (TIF) [file pbio.3000277.s005.tif]

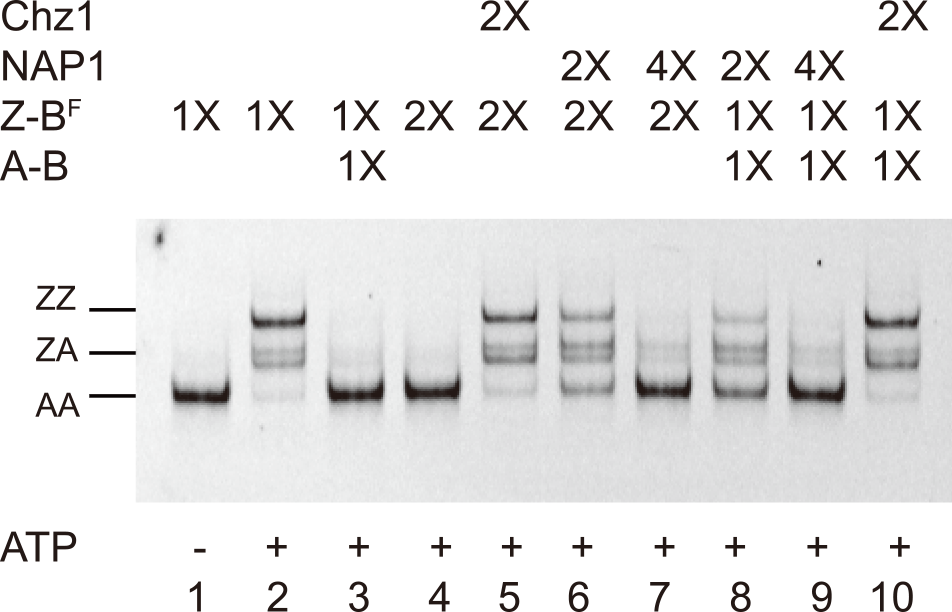

Supplement: S6 Fig — The original gel of Fig 4C. Z-BF refers to Z-B dimer in which the H2B contains a FLAG tag. Chz1, chaperone for H2A.Z-H2B; Nap1, nucleosome assembly protein 1; SWR1, Swi2/snif2-related 1; Z-B, H2A.Z-H2B. (TIF) [file pbio.3000277.s006.tif]
